# Functional Health in Korean Middle‐Aged Women with Poor Sleep Quality

**DOI:** 10.1111/2047-3095.12275

**Published:** 2020-02-17

**Authors:** Go‐Un Kim, Suin Park, Sunah Kim

**Affiliations:** ^1^ Go‐Un Kim, and Sunah Kim are from College of Nursing and Mo‐Im Kim Nursing Research Institute Yonsei University Seoul Republic of Korea; ^2^ Suin Park are from College of Nursing Kosin University Busan Republic of Korea

**Keywords:** Childhood trauma, menopause, middle‐age, post‐traumatic stress disorder, sleep, women's health

## Abstract

**PURPOSE:**

To develop and test a structural model of functional health in middle‐aged women based on the theory of unpleasant symptoms.

**METHODS:**

The direct and indirect effects of menopause status, childhood trauma, post‐traumatic stress, social support, and sleep quality on functional health of 264 Korean women were examined.

**FINDINGS:**

Menopause status and poor sleep quality had a negative direct effect and social support had a positive direct effect on functional health. Menopause status had a negative indirect effect on functional health through poor sleep quality.

**CONCLUSIONS:**

Biopsychosocial nursing intervention programs need to be developed to improve functional health in middle‐aged women.

**IMPLICATIONS FOR NURSING PRACTICE:**

It is important to mediate quality of sleep to improve functional health in middle‐aged women.

**목적**

불쾌증상 이론을 바탕으로 중년 여성의 건강기능을 평가하기 위함이다.

**방법**

264명의 한국 여성을 대상으로 건강기능에 영향을 미치는 폐경, 아동기 외상, 외상 후 스트레스, 사회적 지지의 직ㆍ간접적 영향을 확인하였다.

**결과**

폐경과 낮은 수면의 질은 건강기능에 부적 영향을 미쳤으며, 사회적 지지는 건강기능에 정적 영향을 미쳤다. 폐경은 건강기능에 간접적 영향을 미쳤다.

**결론**

중년여성의 건강기능을 증진하기 위해 신체ㆍ심리ㆍ사회적 요인을 고려한 통합적 중재가 필요하다.

간호실무 적용점: 중년여성의 건강기능을 유지하기 위해 수면의 질을 중재하는 것이 중요하다.

Functional health is a comprehensive concept which indicates whether optimal levels of physiological, psychosocial, and spiritual functions are maintained in individuals, families, and communities in terms of holistic care (Gordon, 1994). Middle‐aged women are in a transitional period during the life cycle between young adults and old age. They experience physical and psychological changes, such as menopause, due to hormonal imbalances. Menopausal symptoms include hot flashes, night sweats, vaginal dryness, and sleep disturbances, as well as health problems, such as obesity, diabetes, cardiovascular disease, and osteoporosis, which lead to both physical pain and psychological problems such as depression (Al‐Safi & Santoro, 2014; Lobo et al., 2014). However, one study found that scores for the self‐management of menopause were 45.60 for premenopausal women and 48.75 for postmenopausal women out of a possible 63 points, which were not high (Kim, Choi, & Kim, 2012). Therefore, it is necessary to confirm the relationship between menopause and the functional health of middle‐aged women in Korea and to provide suggestions regarding the methods of self‐care required to achieve optimal health.

It is important to understand experiences of psychological trauma when dealing with women's health problems. An online survey of 362 middle‐aged Korean women found that they experienced traumatic events due to death (*n* = 93, 25.7%), illness (*n* = 35, 9.7%), breakdown of interpersonal relationships (*n* = 35, 9.7%), and other negative experiences (Jo & Jeong, 2017). Psychological trauma can affect not only mental health but also overall health status, leading to higher rates of morbidity and mortality (Mersky, Topitzes, & Reynolds, 2013). Even childhood trauma experiences have been reported to be a risk factor for sleep disturbances in adulthood (Kajeepeta, Gelaye, Jackson, & Williams, 2015), and they can lead to chronic diseases, such as heart and pulmonary disease, stroke, liver disease, and autoimmune disease, among others (Felitti & Anda, 2010). It has been reported that women are twice as likely to develop post‐traumatic stress symptoms as men (Olff, Langeland, Draijer, & Gersons, 2007). Psychological trauma can be treated with adequate empathy and care through sufficient social support (Laaser, Putney, Bundick, Delmonico, & Griffin, 2017). Therefore, in order to obtain a better understanding of the health status of middle‐aged women, it is necessary to confirm the relationship between health function and experiences of psychological trauma.

Poor sleep quality is one of the major health problems experienced by middle‐aged women. According to the Korea Health Insurance Review & Assessment Service (2017), the proportion of women experiencing poor quality of sleep was 1.5 times higher than that of men. In addition, patients in their 50s had the highest rates of sleep disturbances at 19.9%, followed by 16% for those in their 40s; women were more likely to experience difficulties due to sleep problems than men. When sleep disorders are present, the risk of health problems which leads to poor quality of daily activities, such as anxiety, behavioral problems, cognitive impairment, reduced pain tolerance, overeating, and obesity, increases (Benedict et al., 2011; Penev, 2012). Much of the research into the health of middle‐aged women in Korea is very localized and has focused on specific health problems and conditions such as postmenopausal symptoms and coping strategies, osteoporosis, and cardiovascular disease (Choi et al., 2010; Ham, Kim, Lee, & Choi, 2017). Thus, there is a need to improve the overall functional health of middle‐aged women by providing family and social support.

The theory of unpleasant symptoms (TOUS) can help to explain how quality of sleep is one of the major health problems that has a detrimental effect on the functional health of middle‐aged women. The TOUS, developed by Lenz, Pugh, Milligan, Gift, and Suppe (1997), includes both single and multiple symptom experiences and explains the relationship between performance, which is the result of symptom experience, and the antecedent factors that influence symptom experience, as a dynamic relationship. The conceptual framework of the present study is based on the TOUS (Lenz et al., 1997), and in this study, the direct and indirect factors that are hypothesized to explain functional health are menopausal status, childhood trauma, post‐traumatic stress, and social support. Poor sleep quality is proposed as a direct factor.

## Purpose

The purpose of this study was to construct a theoretical model to explain and predict the functional health of middle‐aged women based on the TOUS (Lenz et al., 1997). The specific aims were to: (a) develop a hypothetical model that explains functional health in middle‐aged women; and (b) construct and verify the functional health model for middle‐aged women with poor sleep quality. The findings of this study will contribute to the knowledge base needed to develop functional health interventions based on a nursing practice rationale.

## Methods

### Design

We conducted a cross‐sectional study utilizing structural equation modeling based on the middle‐range TOUS and constructed a hypothetical model of factors that affect functional health in middle‐aged women. We used the model to verify the data's goodness‐of‐fit and to test our research hypotheses.

### Sample

A total of 500 middle‐aged women completed an online survey and data from 264 participants who were poor sleepers were analyzed. Inclusion criteria were: (a) being a woman aged 40 to 65 years, (b) being able to fully understand the purpose of the study and voluntarily agree to participate in the research, (c) ability to understand the survey questions and respond, (d) having access to the Internet and e‐mail, and (e) being a poor sleeper with a sleep quality score of 5 or more out of a possible 21.

### Main Research Variables

#### Functional health

The Functional Health Pattern Assessment Screening Tool (FHPAST) was developed by Jones (2002) and adapted by Keum and Kim (2012) for use with Korean samples. The FHPAST is a self‐report scale consisting of 58 items, each rated on a 4‐point scale (1 to 4). Sixteen questions (43–58) are reverse scored. Mean scores of three or higher indicate that the physiological‐spiritual function is at a healthy level and the individual is ready for health promotion. The Cronbach's α in Jones's (2002) study was 0.94 and in our study, it was 0.94 as well.

#### Sleep quality

Sleep quality was assessed with the Pittsburgh Sleep Quality Index (PSQI) developed by Buysse, Reynolds, Monk, Berman, and Kupfer (1989) and adapted for Korean use (PSQI‐K) by Sohn, Kim, Lee, and Cho (2012). The PSQI is a self‐report measure of sleep quality over the past month. It contains 22 items, and the total score ranges from 0 to 21. Higher scores indicate lower sleep quality, with scores above five indicating poor quality of sleep (Buysse et al., 1989). We also asked additional questions about sleep quality to identify characteristics related to sleep problems. The Cronbach's α in Sohn et al. (2012) study was 0.84 and, in our study, it was 0.67.

#### Menopausal status

The Menopausal Rating Scale (MRS) was developed by Heinemann, Potthoff, and Schneider (2003) and adapted for Korean use by Choi et al. (2010). The MRS is a self‐report measure that assesses menopausal symptoms and the level of discomfort they cause. This tool is divided into three subdomains (psychological, somatovegetative, and urogenital) and has a total of 11 items. Each item is measured on a 5‐point scale, ranging from 0 to 4 points. Higher scores indicate more severe menopausal symptoms. Symptom severity was categorized by the total score and ranged from none to little (0–4), mild (5–8), moderate (9–16), and severe (17 and above; Heinemann et al., 2003). The Cronbach's α in Heinemann et al. (2003) study was 0.83 and, in our study, it was 0.86.

#### Childhood trauma

The Childhood Trauma Questionnaire (CTQ) was developed by Bernstein and Fink (1998) and adapted for Korean use by Lee (2006). The CTQ is a self‐report scale that identifies a history of childhood trauma experiences in adults. It contains 18 items, and the total score ranges from 0 to 4. Higher scores indicate higher levels of childhood trauma. The Cronbach's α in Bernstein and Fink's study (1998) was 0.90 and, in our study, it was also 0.90.

#### Post‐traumatic stress

The Impact of Event Scale (IES) was developed by Horowitz, Wilner, and Alvarez (1979) and revised by Marmar, Metzler, and Otte (1997). We used a Korean Version of the Impact of Event Scale‐Revised (IES‐R‐K) tool developed by Eun et al. (2005). The tool consists of 22 items and is divided into three subareas (hyperarousal, avoidance, and intrusion), with each area scored on a scale from 0 to 4. Higher scores indicate more severe symptoms. Marmar et al. (1997) reported a Cronbach's α of 0.79; in our study, the Cronbach's α was 0.96.

#### Social support

The Social Support Scale, developed by Park (1985) for Korean research, is a self‐report measure that identifies the degree of support from family members and others when experiencing problems or stress in daily life. It consists of 25 items, divided into four subareas (emotional, informational, material, and valuable). Each area is scored on a scale of 1 to 5, and higher scores indicate higher levels of social support. In Park's (1985) study, the Cronbach's α was 0.94; the Cronbach's *α* was 0.98 in our study.

### Data Collection and Ethical Considerations

We received approval from the Institutional Review Board of our institution (IRB No. Y‐2018‐0033) before data were collected. The data collection period was from April to May 2018. Data were collected via online and mobile phone questionnaires distributed through a commercial online questionnaire agency that has constructed and operated survey panels for over 500,000 people. The initial survey screen explained the objectives and procedures of the research, the data collection process, confidentiality safeguards, and the right for participants to withdraw from the study and have their data excluded without consequence. We also included a letter of commitment to the participants that we would not disclose her personal information for any reason. The questionnaire was only initiated after the participant indicated consent to participate. After completion of the questionnaire, participants’ data were coded to ensure their anonymity. The collected data were used only for research purposes and were stored under restricted access to maintain the anonymity and confidentiality of participants.

### Statistical Analysis

The data were analyzed using IBM SPSS Statistics for Windows, version 23.0 and AMOS version 22. Descriptive statistics were used for general characteristics and measurement variables. Structural models were analyzed as follows: first, normality was examined through skewness and kurtosis. Second, covariance structure analysis was conducted using the maximum likelihood method. Third, *χ* ^2^, χ^2^/*df*, goodness of fit index (GFI), comparative fit index (CFI), Tucker‐Lewis index (TLI), normal fit index (NFI), standardized root mean square residual (SRMR), and root mean square error of approximation (RMSEA) were analyzed to confirm the model fit. Third, to obtain robust results, bootstrapping with 5,000 replications was used to verify the direct and indirect effects on the structural model path. Control variables were age, religion, marital status, education, occupation, drinking alcohol, smoking, exercise, and medication on functional health.

## Findings

### General Characteristics of the Sample

The participants’ mean age was 51.95 ± 7.15 years, and 81.8% (*n* = 216) were married. About 60% (*n* = 158) were affiliated with a religion, 66.7% (*n* = 176) had a college education, and 59.1% (*n* = 156) were employed. Over one‐third (38.3%) of participants reported that they exercised regularly. Over one‐third (39.4%) reported that they drank alcohol at least once a week, and 31.1% reported taking medication because of chronic illness (Table 1).

**Table 1:** General Characteristics of Participants who Reported Poor Sleep (*N* = 264)

Variables	Categories	*N*	%
Age (years)	40–49	97	36.7
	50–59	111	42.0
	60–65	56	21.2
Religion	Yes	158	59.8
	No	106	40.2
Marital status	Unmarried	17	6.4
	Married	216	81.8
	Separated, widowed, or divorced	31	11.7
Education	≤High school	88	33.3
	≥College	176	66.7
Employed	Yes	156	59.1
	No	108	40.9
Monthly income of family (10,000 won, $8.36)	≤350	81	30.7
	351–550	83	31.4
	551–750	49	18.6
	≥751	51	19.3
Monthly allowance (10,000 won, $8.36)	≤30	161	61.0
	31–60	58	22.0
	≥61	45	17.0
Drinking alcohol (once a week)	Yes	104	39.4
	No	160	60.6
Smoking	Yes	23	8.7
	No	241	91.3
Exercise	Yes	101	38.3
	No	163	61.7
Medication (because of chronic illness)	Yes	82	31.1
	No	182	68.9
Menopausal status	Severe	209	79.2
	Moderate	38	14.4
	Mild	10	3.8
	Little	7	2.7
Reason for poor sleep	Physical condition	58	22.0
	Mental health	53	20.1
	Menopause	50	18.9
	Economic difficulties	41	15.5
	Excessive use of a smart phone	26	9.8
	Child‐rearing burdens	23	8.7
	Conflict with husband	21	8.0
	Past difficult experiences	19	7.2
	Interpersonal conflict with others	17	6.4
	Job‐related work	16	6.1
	Burden of household chores	14	5.3
Attempts to improve sleep	Did not attempt any methods	38	14.4
	Drinking warm drinks and tea	38	14.4
	Exercise, yoga, stretching, or walking	37	14.0
	Pillows to help sleep	34	12.9
	Control the evening bedtime	33	12.5
	Take a bath and foot bath	32	12.1
	Adjust the lighting	25	9.5
	Listen to music	21	8.0
	Aromatherapy	7	2.7

Over one‐half (52.8%; *n* = 264) of the 500 middle‐aged women surveyed reported that they slept poorly. In response to items that asked participants to state why they thought they suffered from poor sleep, physical condition was the most frequent response (22%), followed by mental health (20.1%) and menopause (18.9%). Other reasons given included excessive use of a smart phone (9.8%), child‐rearing burdens (8.7%), conflict with husband (8%), and past difficult experiences (7.2%). In response to items asking what methods they used to improve sleep, 14.4% reported they did not attempt any methods, 14.4% reported drinking warm drinks and tea, 14% engaged in exercise, yoga, stretching, or walking, 12.9% used pillows to help sleep, and 12.5% attempted to control their evening bedtime.

### Descriptive Statistics and Correlations

The mean functional health score was 2.68 ± 0.43. A minimum of 3 points indicates adequate health status, suggesting overall inadequate health status in our sample. The average sleep quality score was 8.08 ± 2.17 and scores above 5 indicate poor sleep quality (Table 2).

**Table 2:** Convergent Validity of Latent Variables in Respondents who Reported Poor Sleep (*N* = 264)

Variables	Range	Mean (*SD*)	Skewness	Kurtosis	AVE	CR
Menopausal status	0–4	2.15 (0.79)	−0.407	−0.464	0.52	0.75
Psychological		2.31 (1.10)	−0.527	−0.570		
Somato‐vegetative		2.36 (0.87)	−0.450	−0.138		
Urogenital		1.64 (0.94)	0.166	−0.383		
Childhood trauma	0–9	3.35 (2.09)	0.698	−0.440		
Post‐traumatic stress	0–4	1.18 (0.74)	0.584	0.114	0.84	0.94
Hyperarousal		1.06 (0.83)	0.918	0.584		
Avoidance		1.27 (0.77)	0.297	−0.276		
Intrusion		1.20 (0.76)	0.710	0.453		
Social support	1–5	3.36 (0.78)	−0.715	0.484	0.91	0.98
Emotional		3.35 (0.82)	−0.758	0.403		
Informational		3.39 (0.81)	−0.742	0.633		
Material		3.35 (0.83)	−0.714	0.305		
Valuable		3.35 (0.80)	−0.570	0.410		
Sleep quality	0–21	8.08 (2.17)	1.359	1.709		
Functional health	1–4	2.68 (0.43)	0.144	−0.588		

AVE, average variance extracted; CR, composite reliability; SD, standard deviation.

The mean overall menopausal symptom score of the women with poor sleep quality was 2.15 ± 0.79. The mean scores for each area were: psychological 2.31 ± 1.10; somatovegetative 2.36 ± 0.87; and urogenital 1.64 ± 0.94 (Table 2). Almost all participants (97.4%) reported experiencing menopausal symptoms, with 79.2% having severe, 14.4% having moderate, and 3.8% having mild symptoms, while 2.6% of the participants reported no menopausal symptoms. The mean childhood trauma score was 3.35 ± 2.09. The mean overall post‐traumatic stress score was 1.18 ± 0.74, with subscale scores of hyperarousal 1.06 ± 0.83, avoidance 1.27 ± 0.77, and intrusion 1.20 ± 0.76. The overall social support mean score was 3.36 ± 0.78 and the dimension of informational support was the highest (3.39 ± 0.81; Table 2).

In the univariate normality test, the skewness range across all measures was −0.76‐1.36 and the kurtosis range was −0.59‐ 1.71. We verified the fit of the structural model confirming normal distribution of the variables. The correlations were analyzed to test for multicollinearity among the variables before hypothesis testing. Generally, when the absolute value of the correlation coefficient between variables is less than 0.8, multicollinearity is not considered a problem (Yu, 2012). The correlation coefficients of all the measurement variables in this study ranged from −0.54 to 0.50, so multicollinearity was not present (Table 3).

**Table 3:** Correlation Coefficients between the Latent Variables in Respondents who Reported Poor Sleep (*N* = 264)

	Menopausal status	Childhood trauma	Post‐traumatic stress	Social support	Sleep quality	Functional health
Menopausal status	1	.30**	.50**	−.27**	.41**	−.54**
Childhood trauma		1	.36**	−.45**	.17**	−.38**
Post‐traumatic stress			1	−.25**	.33**	−.39**
Social support				1	−.24**	.50**
Sleep quality					1	−.41**

^**^ *p*< .001.

### Hypothesis Testing

The structural model results were: *χ* ^2^ = 176.72 (*df* = 125, *p* = .002), χ^2^/*df* = 1.41, GFI = 0.95, CFI = 0.98, TLI = 0.97, NFI = 0.95, SRMR = 0.03 and RMSEA = 0.04. The *χ* ^2^ statistic can be sensitive to sample size and the *χ* ^2^/*df* value is generally considered a better indicator of the goodness of fit (Yu, 2012). These results suggest that the hypothesis model to verify the theoretical model met the goodness of fit criteria.

**Figure 1:**
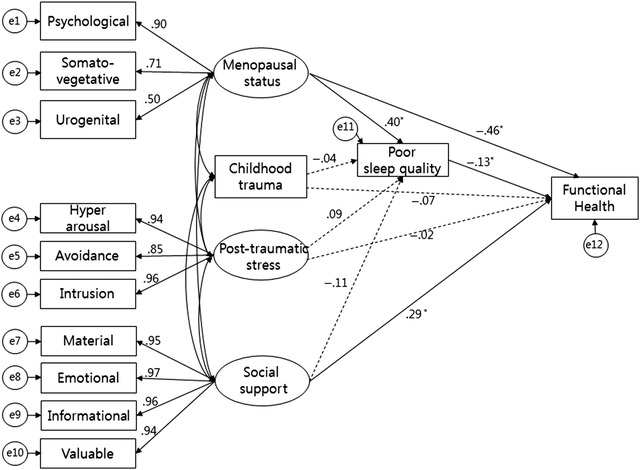
Effect Analysis of Functional Health in Middle‐Aged Women (*N* = 264). Full Lines are Statistically Significant Paths and Dashed Lines are Statistically Nonsignificant Paths. Control Variables were Age, Religion, Marital Status, Education, Occupation, Drinking Alcohol, Smoking, Exercise, And Medication on Functional Health. *p < .05

Menopause status (*β* = −0.46, *p* = .001), social support (*β* = 0.29, *p* = .001), and poor sleep quality (*β* = −0.13, *p* = .041) each had a direct effect on functional health. Menopause status (*β* = 0.40, *p* < .001) had a direct effect on poor sleep quality. Menopause status (*β* = −0.05, *p* = .025) had an indirect effect on functional health through poor sleep quality (Figure 1). The overall explanatory power of the variables influencing functional health was 54.8%.

## Discussion

We developed a hypothetical model for functional health in middle‐aged Korean women and investigated the direct and indirect effects of factors influencing functional health. The results confirmed that functional health is influenced by menopausal symptoms and social support directly, as well as by poor sleep quality.

The study results suggest that functional health and sleep quality in middle‐aged Korean women were not good. The mean functional health score for the middle‐aged women in our sample was 2.68, which is slightly lower than the score reported for a sample of participants with schizophrenia (*M* = 2.70; Keum & Kim, 2012). Of the 500 respondents in this study, 264 (52.8%) experienced sleep problems and poor sleep quality had a direct negative effect on functional health. Previous studies have reported that poor sleep is frequent in middle‐aged women (Tao, Sun, Shao, Li, & Teng, 2016), which is consistent with our results showing that sleep disturbance is common in middle‐aged women. However, 14.4% of participants reported that they passively coped with sleep disturbance (e.g., they did not adopt any methods to improve sleep quality). This finding suggests that individuals with poor quality of sleep and lower levels of functional health might not have adequate information regarding ways to improve sleep or might have received this information but have not acted on it.

Menopausal symptoms had strong, negative direct and indirect effects on functional health and a positive direct effect on poor sleep quality. In addition, considering that the participants of this study were middle‐aged women and that 97.4% of them experienced menopause and 79.2% reported severe menopausal symptoms, it is clear that menopause is the most influential factor on poor sleep quality and functional health. Middle‐aged women in Korea tend to neglect their own health care because they have a negative view of menopause and place an emphasis on important roles such as housework and child rearing (Kim, 2012). However, one study that surveyed the level of knowledge regarding menopause symptom management among 211 Korean middle‐aged women found that knowledge levels were not high: premenopausal women scored 8.60 and postmenopausal women scored 9.17 out of a maximum of 13 points (Kim, Choi, & Kim, 2012). Consequently, the rates of menopausal symptom management may be very low due to low knowledge levels (Kim, Choi, & Kim, 2012); thus, it is necessary to provide education and a support system to improve women's functional health.

Social support was the second most important factor directly affecting functional health in our participants. These results support previous findings that social support exerts a positive influence on health promotion behaviors (Lee, Kim, & Jung, 2014) in middle‐aged women. Social support from family, friends, and colleagues is positively associated with the health status of middle‐aged women (Prairie et al., 2015). Social support gained through supportive relationships may improve an individual's mood and sleep quality (Kent, Uchino, Cribbet, Bowen, & Smith, 2015). Middle‐aged women who have the ability to independently practice self‐care need to strengthen their coping skills to deal with sleep disturbances and to improve their functional health. In this regard, support from their social network and family on the appropriate use of healthcare services might be effective.

Childhood trauma and post‐traumatic stress were not statistically significantly associated with functional health. It is possible that any negative reactions to childhood trauma experienced by the participants diminished and post‐traumatic growth developed over time (Laaser et al., 2017). The results of the World Mental Health Survey reported a stronger effect of childhood physical and sexual abuse, neglect, and parent psychopathology in childhood, adolescence, and early‐to‐middle adulthood than in later adulthood (McLaughlin et al., 2017). However, women may experience a variety of psychological traumas in the workplace such as relationships with co‐workers or supervisors. A survey of lifetime trauma exposure in 1,621 older Korean adults aged 60–74 revealed that 577 (35.6%) experienced trauma at an average age of 30.8 years (Park et al., 2016). The experience of these traumatic events increases the likelihood of developing mental disorders in the elderly (Park et al., 2016). It is important to continuously monitor the elderly so that patients who experience trauma can receive support designed to maintain healthy functioning levels.

This study applied Lenz's et al. (1997) TOUS to identify physiological, psychological, and situational factors affecting overall functional health through poor sleep quality; a typical unpleasant symptom of middle‐aged women is poor sleep quality. It is necessary to assess menopausal symptoms specifically and provide individual and integrative interventions such as hormone and drug therapy and psychological counseling in accordance with the symptoms. In addition, a continuing social support system should be established to improve the overall functional health of middle‐aged women. The physical changes experienced by middle‐aged women due to menopause are difficult to prevent; however, support from family and the community is necessary in order to improve overall functional health of women. There is a need to develop various family‐ and society‐based coping strategies for middle‐aged women in Korea.

## Conclusions

This study developed and verified a theorized model of functional health in middle‐aged women based on the TOUS. The functional health of middle‐aged women was influenced by health‐related factors such as menopausal symptoms, social support, and sleep quality. In order to improve the health of middle‐aged women, it is necessary to have an opportunity to manage health care and improve the management of menopausal symptoms and poor sleep quality. Also, continued support from family and society would help promote the functional health of middle‐aged women. Therefore, interventions aimed at improving the functional health of middle‐aged women need to consider these biopsychosocial aspects.

Given these findings, we suggest the following. First, although this study addressed post‐traumatic stress in middle‐aged women, the effects of childhood trauma on participants was not confirmed directly. It is thus necessary to reconsider the various trauma experiences of middle‐aged women. Second, sleep quality measured using a self‐report questionnaire is subjective and may differ from actual sleep patterns. It is necessary to measure sleep quality objectively using the mobile technology‐based ecological momentary assessments, which might help to monitor their real‐time behaviors and experiences.

## Implications for Nursing Knowledge

This study constructed and verified a theoretical model of functional health in middle‐aged women based on the TOUS.

## Knowledge Translation

Nurses should mediate poor sleep quality to improve functional health among middle‐aged women. In addition, the management of menopausal symptoms and continuous social support are needed to improve the functional health of middle‐aged women using multiprofessional interventions. In particular, it is necessary to develop an intervention plan that takes into account the cultural characteristics of how women perceive menopause.

## Author contributions

SK, SP, and GUK designed the study; SK, SP, and GUK analysed and collected the data; SK and GUK were involved in the manuscript preparation.

## Conflict of interest

The authors have no conflicts of interest to report.
